# The Switch in a Genetic Toggle System with Lévy Noise

**DOI:** 10.1038/srep31505

**Published:** 2016-08-19

**Authors:** Yong Xu, Yongge Li, Hao Zhang, Xiaofan Li, Jürgen Kurths

**Affiliations:** 1 Department of Applied Mathematics, Northwestern Polytechnical University, Xi’an 710072, China; 2 Potsdam Institute for Climate Impact Research, Potsdam 14412, Germany; 3 Department of Applied Mathematics, Illinois Institute of Technology, Chicago, IL 60616, USA

## Abstract

A bistable toggle switch is a paradigmatic model in the field of biology. The dynamics of the system induced by Gaussian noise has been intensively investigated, but Gaussian noise cannot incorporate large bursts typically occurring in real experiments. This paper aims to examine effects of variations from one protein imposed by a non-Gaussian Lévy noise, which is able to describe even large jumps, on the coherent switch and the on/off switch via the steady-state probability density, the joint steady-state probability density, and the mean first passage time. We find that a large burst of one protein due to the Lévy noises can induce coherent switches even with small noise intensities in contrast to the Gaussian case which requires large intensities for this. The influences of the stability index, skewness parameter and noise intensity on the on/off switch are analyzed, leading to an adjustment of the concentrations of both proteins and a decision which stable point to stay most. The mean first passage times show complex effects under Lévy noise, especially the stability index and skewness parameter. Our results also imply that the presence of non-Gaussian Lévy noises has fundamentally changed the escape mechanism in such a system compared with Gaussian noise.

The ability to switch between two states is very important for organisms in biological systems to adapt to different environments^1^,^2^,^3^, such as the Lambda phage switch between lysogenic state and lytic state^4^. A synthetic toggle switch in *E. coli* was first constructed by Gardner *et al*.^5^, consisting of a pair of repressors that repress each other’s expression. The toggle has been analyzed both deterministically and stochastically as it can provide a theoretical prediction of the conditions sufficient for bistability and studies have shown that it can be used as basic module of organisms.

Researches on the toggle switch in the presence of noise have been considered in two aspects: switching and stochastic resonance (SR)^6^,^7^,^8^,^9^,^10^,^11^,^12^,^13^,^14^. For example, Velia^7^ found that a dramatic increase in protein noise level would cause the cell to randomly switch between two states. Tian *et al*.^8^ first added Poisson random numbers to the toggle switch system, and found that Poisson noise could induce switching. Wang *et al*.^9^ applied Gaussian noise to simulate the external environment, and studied the switching behavior under external excitations. They found that a moderate noise intensity can induce the best coherent switch. Munsky *et al*.^10^ adjusted the number of mRNA in an experiment to simulate random fluctuations of the external environment and found that external noises could induce switches. Strasser *et al*.^11^ investigated the switch and the stability of a toggle switch model of stochastic gene expression. In terms of SR, Koseska *et al*.^12^ studied the expansion three-dimensional toggle switch induced by Gaussian noise, and found that noise can control the system’s integrity behavior. Daza *et al*.^13^ studied a toggle switch delay model induced by a double periodic force, and observed the phenomenon of SR. Hellen *et al*.^14^ studied logic stochastic resonance (LSR) of toggle switch induced by Gaussian noise, and realized the LSR.

Stochastic transcriptional burst, a transient transcription of large pulses from DNA to RNA, has been observed in diverse organisms, from bacteria to mammals^15^,^16^,^17^. Scholars have used the Gillespie algorithm to simulate the gene expression process for a long time^18^,^19^, and nowadays Poisson noise or added Poisson factor have been applied into the Gillespie algorithm to model stochastic bursts which seems to be a more reasonable way than small perturbations adopted before^20^,^21^,^22^,^23^. In fact, small perturbations plus stochastic pulses are a simple Lévy noise model whose most important feature is a large jump^24^. Furthermore, the skewness parameter enables Lévy noise to produce an asymmetric noise distribution, which takes a key role on phenomena that transitions between stable points occur frequently in a noisy field, while Gaussian white noise can hardly induce this kind of translations due to its symmetric distribution. Comparing to Gaussian noise, a Lévy distribution is a more appropriate choice when one considers realistic models with pulse phenomena in various systems^25^,^26^,^27^,^28^,^29^,^30^,^31^. For example, by analyzing experimental data we have recently found Lévy noise in a Laser Gyroscope differing from the previous finding that divides the noises into several parts^27^. Kong *et al*.^31^ proved that the data of evoked potential obeys a Lévy distribution, which indicates the existence of alpha-stable noise in a biological system. Recently, effects of non-Gaussian Lévy noise on nonlinear systems have attracted growing attentions in physics, biology, engineering, natural science and social science^32^,^33^. In our previous work^34^, we have examined a simple case of single gene expression, which is the fundamental module of gene regulatory circuits and contains the essential features of transcription and translation. We found that Lévy noise can induce a bistable genetic switch and can influence the mean first passage time (MFPT) which are rather different from the Gaussian case.

In this paper, we mainly focus our interest on Lévy fluctuations on one protein and evaluate the role of Lévy noise in the genetic toggle switch model consisting of two protein repressor genes and two promoters. We will show that there are fundamental differences in the switch behavior between non-Gaussian Lévy noise and Gaussian noise. We find in particular that small Lévy noise intensities can induce switches between the stable states in this bistable system.

## Materials and Methods

### Genetic toggle switch system with Lévy noise

**Figure 1:**
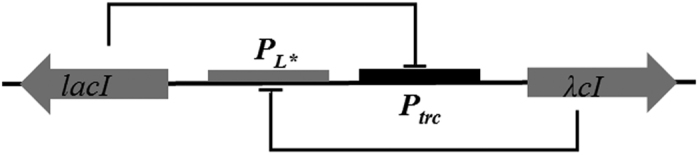
The toggle switch design5,6. In our case, the regulatory circuit is comprised of two genes, *lacI* and *λcI*, that encode the transcriptional regulator proteins, *LacI* and *λCI*. Gene *lacI* denotes the *Lac* repressor in conjunction with the *Ptrc* promoter and *λcI* represents the temperature-sensitive repressor in conjunction with the *P*_L*_ promoter.

As shown in Fig. 1, the promoter that controls the expression of *λcI* is attenuated by the protein *LacI,* and the promoter that controls the expression of *lacI* is attenuated by the protein *λCI*. Thus, two states are possible: *λCI* is abundant and *LacI* is scare (the *λCI*-on state) or *LacI* is abundant and *λCI* is scare (the *LacI*-on state). The deterministic behavior of the toggle switch can be described by the following equations^5^:


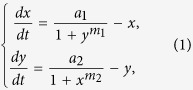


where *x* and *y* are the concentrations of *LacI* and *λCI* respectively, *a*_1_ and *a*_2_ represent the dimensionless transcription rates in the absence of the repressors, and *m*_1_ and *m*_2_ are the Hill coefficients.

**Figure 2:**
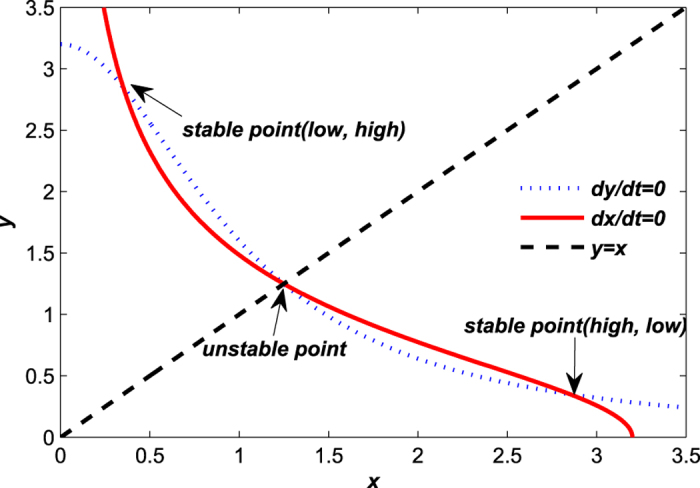
Geometric structure of the toggle equations (1). The line *dx*/*dt* = 0 (red) and the line *dy*/*dt* = 0 (blue) intersect at three points: two stable points (*x*_*low*_ = 0.351, *y*_*high*_ = 2.849), (*x*_*high*_ = 2.849, *y*_*low*_ = 0.351) and one unstable point (1.2495, 1.2495), indicating that the system is bistable with the parameters of the system (1) given by *a*_1_ = *a*_2_ = 3.2, *m*_1_ = *m*_2_ = 2.0.

Figure 2 shows the bistablity of the toggle switch system (1)^5^,^8^,^35^. The two stable points (*x*_*low*_, *y*_*high*_) and (*x*_*high*_, *y*_*low*_) are called (*low, high*) state (the *LacI* protein in low state and the *λCI* protrein in high state) and (*high, low*) state (the *LacI* protein in high state and the *λCI* protein in low state), where the (*low, high*) state means the *LacI* protein is repressed and the *λCI* protein is activated while the (*high, low*) state is the opposite.

**Figure 3:**
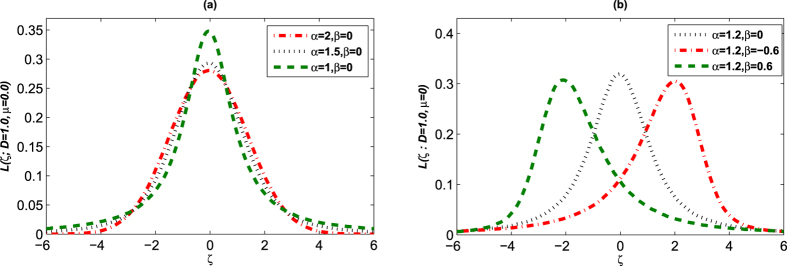
The PDFs for different parameters with *μ* *=* 0. (**a**) When *β* = 0, the distributions are symmetric, and decreasing *α* makes the distributions slimmer and higher with heavy tails. When *α* = 2, it becomes the Gaussian distribution. (**b**) For fixed *α* > 1, when *β* < 0, the distribution is right-skewed, when *β* > 0, it becomes left*-* skewed.

Noise-induced switches have been observed widely in biological systems^36^, especially in bistable systems between the two stable wells. In particular, there are powerful evidences indicating that Lévy noises exist in biological environments abundantly^31^. It has been shown clearly that gene expression experiences the noisy environment, containing small diffusion and large bursting, which likes the composition of Lévy noise. Basing on the previous work on the toggle under Gaussian noise, we extend the work to the Lévy case. As far as we know, this is the first attempt to model gene expression by Lévy noise, to investigate possible different effects of Lévy noise compared with Gaussian noise. We put our objective on one-protein fluctuations induced switch and suppose the noise imposed on another protein to be zero. So in this paper, we construct the following stochastic model of gene regulatory system by adding a Lévy noise term to the deterministic system (1):


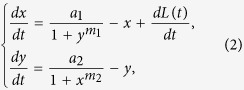


where *L*(*t*) is a Lévy stable motion with stationary and independent increments on non-overlapping time intervals. Lévy noise, as the formal derivative of Lévy motion, is able to characterize the appearance of large jumps, which benefits from its quadruple distribution *L*(*ζ: α, β, c, μ*) described by the characteristic function *ϕ*(*θ*),


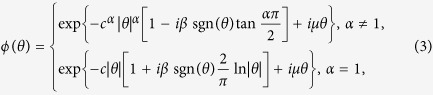


where *α*(0 < *α* ≤ 2) is called the stability index, determining the thickness of the tail (the distribution is Gaussian for *α* = 2), *β*(−1 ≤ *β* ≤ 1) is the skewness parameter to measure the symmetry of the system, *c* is the scale parameter and *D* = *c*^*α*^ denotes the noise intensity, and *μ* is the mean of the distribution. The distribution is symmetric about *μ* for *β* = 0. Probability density functions (PDFs) of the Lévy distribution are illustrated in Fig. 3 with different values of the parameters.

**Figure 4:**
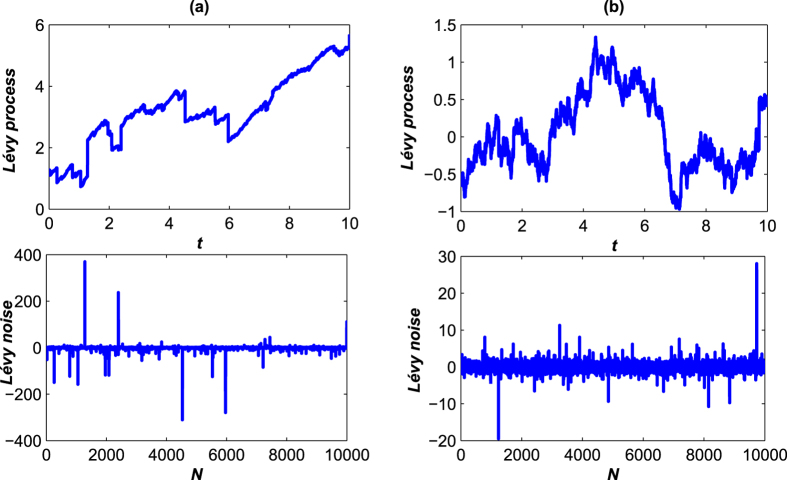
The trajectories of Lévy process and Lévy noise with different parameters. (**a**) The diagrams of Lévy process and the corresponding Lévy noise with 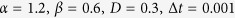 are plotted; (**b**) the diagrams of Lévy process and the corresponding Lévy noise with *α* = 1.8, *β* = 0, *D* = 0.1, Δ*t* = 0.001 are plotted.

Based on the self-similarity of Lévy process, for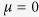 (which holds in the following context, because if *μ* ≠ 0 we can make a simple transform on the system to make it zero), we get the relation^37^


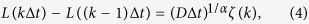


where *ζ*(*k*) is the time-discrete Lévy noise whose characteristic function is given by *L*(*t*) with *t* = 1, *D* = 1^38^. Figure 4 shows the Lévy process and Lévy noise for different parameters.

## Results and Discussions

In this part, we will discuss both the coherent switch and the on/off switch for the toggle switch model (2). The coherent switch induced by noise describes the transitions between two states, which can be observed from the sample path. This implies that the gene expression samples change from staying in only one state ((*high, low*) state or (*low, high*) state) to switching between both states. From the point of joint steady-state probability density (JSPD) of the protein concentrations, the coherent switch happens when probabilities are greater than zero in both states. On the other hand, the on/off switch is measured by the JSPDs, which requires that the probability value of the low state increase and overtake that of the high state. The switch is at the state of ‘off’ when the concentration is at the low state, conversely, called ‘on’ when the expression of the high state is excited^35^. We will first consider the coherent switch and then the on/off switch.

### Noise-induced coherent switch

Noise can induce switches in bistable systems^39^,^40^, however, the phenomena vary a lot when the system is subject to different kinds of noises^34^,^41^. As the Lévy distribution possesses heavy tails and discontinuous jumps^25^,^26^,^27^,^28^, which well coincides with properties of real gene expression noise, we intend to study the switch in (2) induced by Lévy noise, and analyze how the system responds to tempestuous stimuli.

**Figure 5:**
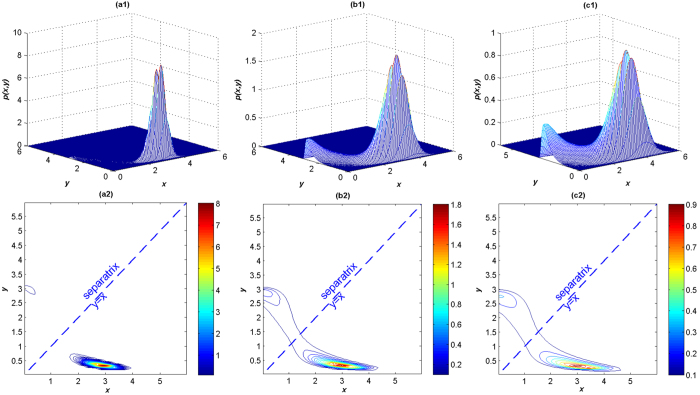
The JSPDs and corresponding contour plots of the *LacI* and the λCI proteins under Gaussian case *α* *=* 2. (**a**) *D* = 0.1, *β* = 0.5; (**b**) *D* = 0.3, *β* = 0.5; (**c**) *D* = 0.5, *β* = 0.5.

In order to get a negative feedback, we set *m*_1_ = *m*_2_ = 2.0 and *a*_1_ = *a*_2_ = 3.2, and assume the initial concentrations of the proteins as *x*(0) = 3.2 and *y*(0) = 0.3. We keep these values of the parameters in our calculations of this paper. First, let us consider the Gaussian case when *α* = 2.0. Using standard numerical methods^42^, we get the JSPDs of *LacI* and *λCI* shown in Fig. 5 from which we can find the probability distributions of the *LacI* and *λCI* proteins in both states under different noise intensities. In Fig. 5(a), we see that only a tiny probability of the *LacI* protein exists in the (*low, high*) state, while most time the protein stays in the (*high, low*) state (the difference between nearest-neighbor lines in the contour figures is Δ*h* = 0.1 which holds in the full paper), which means when *D* = 0.1 the noise can hardly induce a switch between the two stable states, and the coherent switch almost can not occur when induced by Gaussian noise with weak noise intensities. With the increase of noise intensity, when *D* = 0.3, the probability slides slowly from the (*high, low*) state to the (*low, high*) state leading to an enhancement of probability in the area of (*low, high*) state. This suggests that the system stays at the (*high, low*) state most of the time and occasionally switches to the other stable state. When *D* = 0.5, the distribution is diffused indicating that the switch occurs frequently, but the system always prefers to stay in the (*high, low*) state. We observe that moderate and strong Gaussian noise intensities can induce coherent switch, while weak noise intensities can hardly induce switches as shown in Fig. 5. The result agrees with that obtained by Yuan *et al*.^43^.

As the Gaussian noise only acts on the *LacI* protein and the system is mutual inhibition, we observe that the peak of the (*high, low*) state is always larger than that of the (*low, high*) state no matter how the intensity increases. The phenomenon can be explained as: the left stable point is (*x*_*low*_ = 0.351, *y*_*high*_ = 2.849) and the right (*x*_*high*_ = 2.849, *y*_*low*_ = 0.351), when adding symmetric Gaussian noise to *x* in the (*high, low*) state, if it wants to switch to the (*low, high*) state, *x* has to cross the unstable point (1.2495, 1.2495) to the other side of the sparatrix, that is to say, *x*_*high*_ = 2.849 should shift Δ*x*_*h*→*l*_ = *x*_*high*_ − *x*_*unstable*_ = 1.5595. However, if it wants to switch from (*low, high*) state to (*high, low*) state, *x*_*low*_ should be kicked by an excitation at least 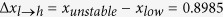. So we see that it is much easier to transfer from the (*low, high*) state to (*high, low*) state, and this is why the right peak always dominates.

**Figure 6:**
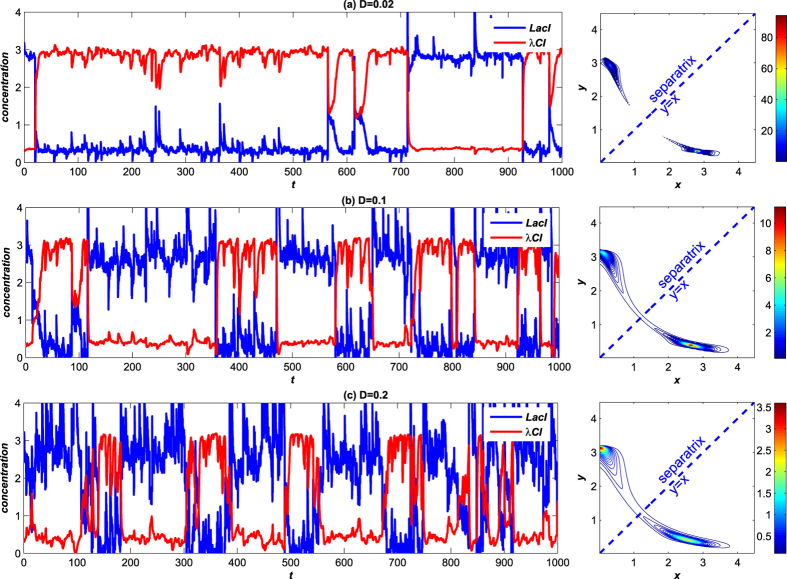
The sample paths under the Lévy noise. The blue and the red lines represent the *LacI* and *λCI* proteins concentrations respectively. (**a**) *α* = 1.2, *β* = 0.3, *D* = 0.02; (**b**) *α* = 1.2, *β* = 0.3, *D* = 0.1; (**c**) *α* = 1.2, *β* = 0.3, *D* = 0.2.

Next, we consider non-Gaussian Lévy noise by setting *α* = 1.2. The sample paths with different noise intensities are plotted in Fig. 6. We observe that the coherent switch occurs not only at moderate (*D* = 0.2) but also at weak noise (*D* = 0.02 and *D* = 0.1), in contrast to the Gaussian noise case presented above.

### Noise-induced on/off switch

The steady-state probability density (SPD) is one of the best ways to describe the dynamical behaviors in bistable systems^11^,^34^,^35^, including the on/off switch. In noise-induced bistable systems, the concentration of a protein will probably change between two stable states, thus the SPDs may concentrate on the stable points with two peaks. Based on the probability value around the two states, it is called ‘on’ state if the concentration stays more on the high state in which the expression of the gene in the low state is suppressed, otherwise it is called ‘off’ where the expression in the high state is suppressed^35^.

**Figure 7:**
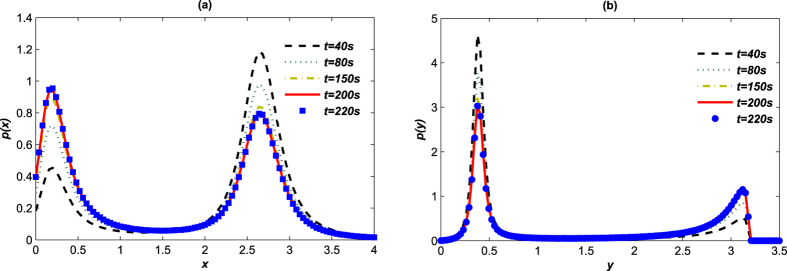
The PDFs at different time. The PDFs for *LacI* (**a**) and *λCI* (**b**) proteins concentrations with *α* = 1.2, *β* = 0.3.*D* = 0.1, *μ* = 0, The sample size is 1 × 10^7^.

To investigate the steady-state response, the steady-state time should be obtained first. Plenty of sample paths have been calculated to generate the PDFs at different time. If the probability density stays the same after a specific time, the value of the time is considered to be in steady-state regime. In Fig. 7 marginal SPDs of the toggle switch system (2) with Lévy noise stay almost the same around *t* = 220 *s*, which will be regarded as the steady-state time in all this paper’s calculations.

Now, the influences of each parameter of Lévy noise on gene switch will be discussed in detail that is ‘on’ to ‘off’ switch or ‘off’ to ‘on’ switch.

**Figure 8:**
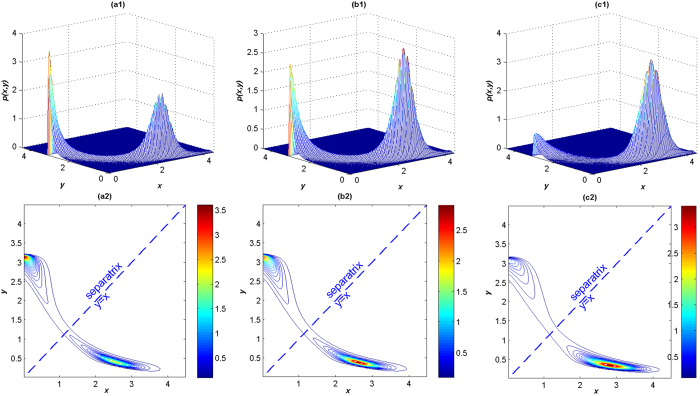
The JSPDs and contour plots of *LacI* protein and *λCI* protein for different values of *α*. (a1, a2) *α* = 1.2, *β* = 0.3, *D* = 0.2; (b1, b2) *α* = 1.27, *β* = 0.3, *D* = 0.2; (c1, c2) *α* = 1.6, *β* = 0.3, *D* = 0.2.

First, we discuss the influence of the parameter *α*. By numerical simulation of system (2), the JSPDs of the *LacI* protein and the *λCI* protein are shown in Fig. 8. The corresponding contour plots of the JSPDs list together. From the JSPDs, we find that the two proteins are in mutual inhibition. The proteins are in the (*low, high*) state when *α* = 1.2 as clearly shown in the JSPD in Fig. 8(a1). The *LacI* protein is in the ‘off’ state, and the *λCI* protein is in the ‘on’ state, which indicates the expression of (*high, low*) state is suppressed. With the increase of *α*, the peak of the JSPD at (*high, low*) state increases in height and finally exceeds the other peak as *α* exceeds a critical value which is approximately 1.27(Fig. 8(b1)). In this case the system stays in a small neighborhood of two stable points with about the same probability. The *LacI* protein and the *λCI* protein have almost the same opportunity for the ‘on’ or ‘off’ state. Continuing to increase *α* to 1.6, as shown in Fig. 8(c1), we find that the system spends more time on the *LacI* protein ‘on’ state and the *λCI* protein ‘off’ state. Based on the contour plots, we see that the two peaks are separated by the line *y* = *x*, with the increase of *α*, the probability in the (*low, high*) state slides to the (*high, low*) state by crossing the separatrix around the unstable point (1.2495, 1.2495) confined in a narrow area. When *α* is large enough, most probability is transported to the (*high, low*) point which accomplishes the process of switching from one state to the other. These results imply that increasing *α* can induce the switch from (*high, low*) state to (*low, high*) state when other parameters are fixed. As different kinds of noises can be represented by different values of *α*, the results also provide compelling evidence that there is a noticeable difference between the dynamics of the system under Gaussian and non-Gaussian Lévy noises.

**Figure 9:**
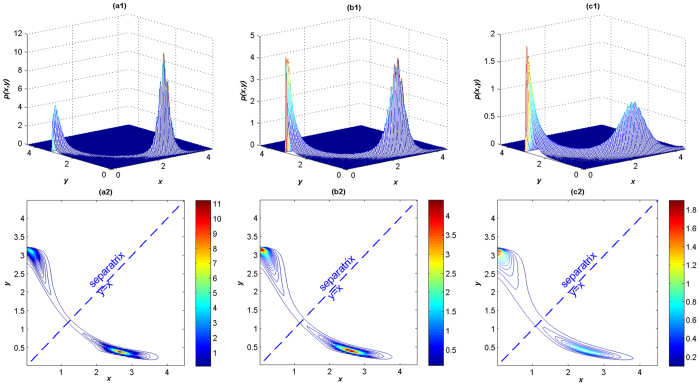
The JSPDs and corresponding contour plots of *LacI* and *λCI* proteins for different values of *D*. (a1, a2) *α* = 1.2, *β* = 0.3, *D* = 0.1; (b1, b2) *α* = 1.2, *β* = 0.3, *D* = 0.15; (c1, c2) *α* = 1.2, *β* = 0.3, *D* = 0.3.

Second, we study the influence of the noise intensity *D*. The JSPDs and contour plots of *LacI* and *λCI* proteins with different noise intensities are presented in Fig. 9. In Fig. 9(a1), the peak of the JSPD at the (*high, low*) state is much higher when *D* is small, thus the *LacI* protein is in ‘on’ state, while the *λCI* protein is in ‘off’ state on the contrary. Unlike the previous contour plots of the JSPD, the contour plots in Fig. 9(a2) show opposite distributions when *D* = 0.1. Increasing *D*, the protein level at the (*low, high*) state becomes higher gradually. When *D* exceeds the critical value that the two peaks in the JSPD share the same heights, around *D* = 0.15, the peak value of the (*low, high*) state overtakes that of the (*high, low*) state, which switches the *LacI* protein from the suppressed state to the excited one, while *λCI* protein performs the opposite transition. Compared with Fig. 5, we find that with the same intensity, Lévy noise plays a total different influence on the behaviors of the toggle switch system, that the Gaussian noise can’t induce switch. The JSPDs in the Lévy case are much more slim and sensitive. Hence, Lévy noise is able to realize the on/off switch easily.

**Figure 10:**
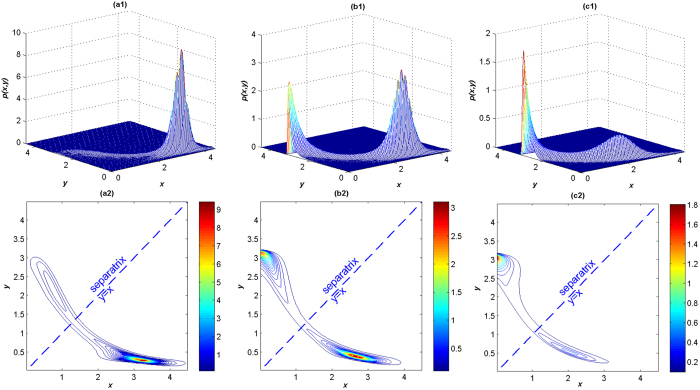
The JSPDs and corresponding contour plots of *LacI* protein and *λCI* protein for different *β*. (a1, a2)*α* = 1.2, *β* = −0.3, *D* = 0.2 (b1, b2) *α* = 1.2, *β* = 0.2, *D* = 0.2 (c1, c2) *α* = 1.2, *β* = 0.8, *D* = 0.2.

Finally, the influence of the skewness parameter *β* in the Lévy noise is studied. We calculate the JSPDs of *LacI* and *λCI* proteins with different values of *β* in Fig. 10. When *β* = −0.3, the probability focuses on the (*high, low*) state, leaving merely a little for (*low, high*) state. Therefore, the *LacI* protein is in the ‘on’ state and the *λCI* protein is in the ‘off’ state. Increasing *β*, the right peak of the JSPD becomes lower gradually. When *β* = 0.2, the peak of (*low, high*) state has almost the same height with that of the (*high, low*) state. Upto *β* = 0.8, the JSPD realizes a total inversion, that most of the probability transfers from (*low, high*) state to (*high, low*) state, implying that *β* can induce switch from the (*high, low*) state to the (*low, high*) state.

Different from the stability index *α* and noise intensity *D*, we find in the contour plots in Fig. 10 that the skewness parameter shifts the positions of the peaks in JSPDs obviously. In fact, for *β* > 0, the distribution of Lévy noise is left-skewed, while for *β* < 0, it is right-skewed. When we add left-skewed Lévy noises to *x*, it will make *x* smaller in the mean sense, so under left-skewed noises (*β* > 0), we find (*high, low*) point shifts to the left, while with right-skewed noises (*β* < 0), the (*high, low*) point shifts to the right.

### Mean first passage time

The previous analysis indicates that the parameters of Lévy noise can strongly affect the switch of a gene. But how long will it spend to switch from low to high concentration and vice versa is another interesting issue for concern, which is referred to the mean first passage time^44^.

In recent years, conventional problems of the escape over the fluctuating potential barrier have attracted a great deal of attention^44^,^45^,^46^,^47^,^48^,^49^,^50^. For bistable or multistable systems, the switch between different states may occur when induced by noises. The MFPT is used to measure the time interval how long will the system take to switch from one state to another^44^,^45^,^46^,^47^. However, the MFPTs are different when the system is induced by different kinds of noises^48^,^49^.

When the system switches from low to high state, it is called positive MFPT^+^, otherwise called reversed MFPT^−^. Taking the positive MFPT^+^, the value of the first time that the protein switches from the low state to the high state is regarded as the positive first passage time (FPT). Due to the mutual inhibition of two proteins, we only discuss the positive MFPT of the *LacI* and *λCI* proteins: the positive MFPT for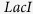 is the reverse MFPT for *λCI* and vice versa.

Since the system is 2-dimensional, the exact determination of the FPT is complex. For practical purposes, in order to determine the MFPT from (*low, high*) state to (*high, low*) state, we assume the following definition of the FPT as follows:


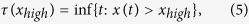


in which the variable *y* is not necessary to count. In the above contour plots, due to the Hill function, we see that the probability is restricted in limited region, when *x* is large then *y* will be small, we do not need to consider a 2-dimensional computation. Passage and pseudo-passage are merely counted mixed. And then the MFPT^+^ is defined as:


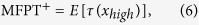


where E[·] is an averaging operator.

Take the positive MFPT^+^ of the *LacI* protein for example, set 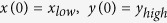, applying the Monte Carlo method for the numerical simulation, let Δ*t* be the time step, if *x*_*high*_ < *x*(*k*Δ*t*), then the *τ*(*x*_*high*_) = *k*Δ*t*. Let the number of simulation times be *N*, then


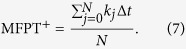


**Figure 11:**
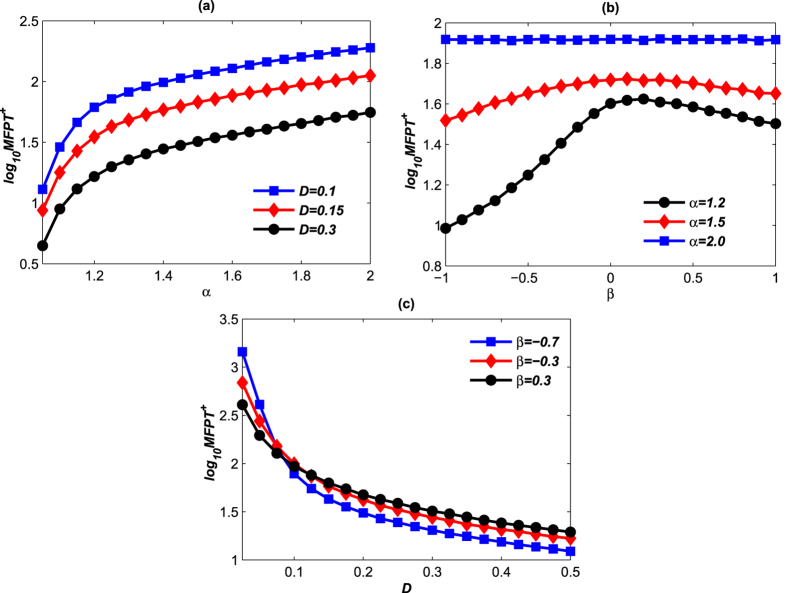
MFPT^+^ of *LacI* protein transferring from the low state *x*_*low*_ to the high state *x*_*high*_. Take the time step Δ*t* = 0.01 and average over *N* = 50000 different noise realizations. (**a**) the MFPT^+^ versus stability index *β* = −0.3; (**b**) MFPT^+^ versus skewness parameter *D* = 0.2; (**c**) MFPT^+^ versus noise intensity *α* = 1.4.

Figure 11 shows the effects of the noise parameters on the positive MFPT of the *LacI* protein. In the simulation, we let the starting point at the low state, and then calculate the average of first passage time over 50000 realizations. The MFPT^+^ versus noise intensity, stability index, and skewness parameter are shown in Fig. 11.

The MFPT^+^ decreases due to the increase of the stability index as shown in Fig. 11(a) for the noise intensities 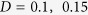 and 0.3. In terms of biological implications, when the *LacI* concentration stays at the low state, the transition from the low concentration state to the high concentration state becomes easier with the decrease of the stability index and increase of *D*. It indicates that a small *α* and a large *D* can speed up the expression of *LacI* protein. Interestingly, it is shown in Fig. 11(b) that the increasing of *β* leads to a non-monotone response: the MFPT^+^ initially increases, reaches a maximum, and then decreases with further increase of *β*. In contrast, *β* has no effect on the system (1) when the noise is Gaussian. In Fig. 11(c), the decreases in MFPT^+^ due to the increase of *D* can be observed. For different *β*, the curves decrease in different speeds. That is with the increase of the *β*, the MFPT^+^ decreases more and more slowly. It indicates that large *D* and small *β* speed up the expression of the *LacI* protein while suppress the expression of the *λCI* protein.

**Figure 12:**
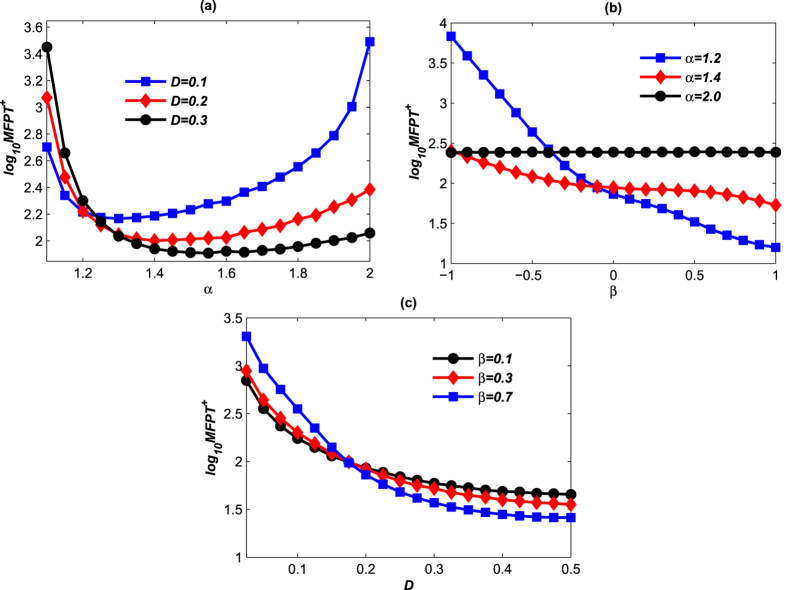
MFPTs^+^ of *λCI* protein of the transition from the low-expression state *y*_*low*_ to the high-expression one *y*_*high*_. All MFPTs^+^ are obtained by taking the time step Δ*t* = 0.01 and averaging over *N* = 50000 different noise realizations. (**a**) MFPT^+^ versus stability index while keeping *β* = −0.3; (**b**) MFPT^+^ versus skewness parameter while keeping *D* = 0.2; (**c**) MFPT^+^ versus noise intensity while keeping *α* = 1.4.

The MFPT^+^ of the *λCI* protein changing from the low to high concentration is presented in Fig. 12. Although the noise acts on the *LacI* protein directly in our model, the mutual inhibition induces switch in the *λCI* protein concentration as shown in Fig. 12(a). The average time decreases first, and then increases with the increase of the stability index. The average time as a function of the stability index is lower convex; thus there is an optimal value of the stability index to reach the minimum value of MFPT^+^ at each level of the noise intensity. Figure 12(b) shows that the smaller the stability index is the faster the MFPT^+^ decreases with the increase of the skewness parameter. We see that the change of MFPT^+^ when *α* = 1.2 is obvious, i.e. the skewness plays an important role in the Lévy noise, while it has no effect in the Gaussian case. As shown in Fig. 12(c), the MFPT^+^ decreases with the increase of the noise intensity. The noise intensity can speed up the expression of *λCI* protein as shown like Fig. 11(c).

In this part, we present how the parameters of Lévy noise affect the MFPT^+^ of both *LacI* and *λCI* protein. Through the MFPT^+^ we get a deep insight into the on/off switch. The results show that the MFPT^+^ and JSPD can both describe the propensity of protein concentration switch in two states and the parameters of Lévy noise can really control the change of protein concentration.

## Conclusions

In this paper we have studied the coherent switch, the on/off switch and MFPT induced by Lévy noise in a Toggle Switch bistable system. Using Monte Carlo methods, the sample paths of the *LacI* and *λCI* protein concentrations are calculated, and switches are observed. For the coherent switch, we find that very small intensities of the non-Gaussian Lévy noise can already induce switches, which is different from the previous finding that only large Gaussian noises can induce a switch. For the on/off switch, we find that the switch from (*low, high*) state to (*high, low*) state occurs when we increase the stability index of the Lévy noise. The noise intensity can drive the switch from (*high, low*) state to (*low, high*) state when it increases. The skewness parameter can also induce a switch when it increases. The expression of the *LacI* protein in high state is more excited when the skewness parameter increases which has no effect in the Gaussian situation. The Lévy noise has larger jumps when the stability index decreases or the noise intensity increases. This implies that worse environments can make the proteins switching between the (*low, high*) state and (*high, low*) state.

We have also studied the MFPT^+^, namely the mean first passage time for a protein to switch from its low state to its high state. With all other parameters fixed, increasing the stability index of the noise will reduce the MFPT^+^ of the *LacI* protein, which agrees with the result in ref. 51. The MFPT^+^ is shorter when the skewness changes from the symmetric case or the noise intensity increases. Our results have demonstrated that the Lévy noise fundamentally changes the switch mechanism of the proposed system.

## Additional Information

**How to cite this article**: Xu, Y. *et al*. The Switch in a Genetic Toggle System with Lévy Noise. *Sci. Rep.* **6**, 31505; doi: 10.1038/srep31505 (2016).
